# Sociobehavioural factors associated with SARS-CoV-2 infection and COVID-19 vaccine effectiveness against medically attended, symptomatic SARS-CoV-2 infection in the Philippines: a prospective case-control study (FASCINATE-P study)

**DOI:** 10.5365/wpsar.2025.16.1.1131

**Published:** 2025-01-15

**Authors:** Takeshi Arashiro, Regina Pascua Berba, Joy Potenciano Calayo, Marie Kris, Reby Marie Garcia, Shuichi Suzuki, Cecile Dungog, Jonathan Rivera, Greco Mark Malijan, Kristal An Agrupis, Mary Jane Salazar, Mary Ann Salazar, Jinho Shin, Martin Hibberd, Koya Ariyoshi, Chris Smith

**Affiliations:** a Faculty of Infectious and Tropical Diseases, London School of Hygiene and Tropical Medicine, London, United Kingdom of Great Britain and Northern Ireland.; b School of Tropical Medicine and Global Health, Nagasaki University, Nagasaki, Japan.; c Center for Surveillance, Immunization, and Epidemiologic Research, National Institute of Infectious Diseases, Tokyo, Japan.; d Department of Pathology, National Institute of Infectious Diseases, Tokyo, Japan.; e World Health Organization Regional Office for the Western Pacific, Manila, Philippines.; f Hospital Infection Control Unit, Philippine General Hospital, Manila, Philippines.; g Department of Laboratory, San Lazaro Hospital, Manila, Philippines.; h San Lazaro Hospital-Nagasaki University Collaborative Research Office and Laboratory, San Lazaro Hospital, Manila, Philippines.; i Department of Laboratories, Philippine General Hospital, Manila, Philippines.

## Abstract

**Objective:**

We examined sociobehavioural factors associated with SARS-CoV-2 infection and estimated COVID-19 vaccine effectiveness against symptomatic SARS-CoV-2 infection in the Philippines. Such studies are limited in low- and middle-income countries, especially in Asia and the Pacific.

**Methods:**

A case-control study was conducted in two hospitals in Manila, Philippines, from March 2022 to June 2023. Sociobehavioural factors and vaccination history were collected. PCR-positive individuals were cases, while PCR-negative individuals were controls. Adjusted odds ratios (aORs) were calculated to examine associations between sociobehavioural factors/vaccination and medically attended SARS-CoV-2 infection.

**Results:**

The analysis included 2489 individuals (574 positive cases, 23.1%; 1915 controls, 76.9%; median age [interquartile range]: 35 [27–51] years). Although education and household income were not associated with infection, being a health-care worker was (aOR: 1.45; 95% confidence interval [CI]: 1.03–2.06). The odds of infection were higher among individuals who attended gatherings of five or more people compared to those who attended smaller gatherings (aOR: 2.58; 95% CI: 1.14–5.83). Absolute vaccine effectiveness for vaccination status was not estimated due to a high risk of bias, for example, unascertained prior infection. Moderate relative vaccine effectiveness for the first booster (32%; 95% CI: −120–79) and the second booster (48%; 95% CI: −23–78) were observed (both with wide CI), albeit with a waning trend after half a year.

**Discussion:**

The higher odds of infection among health-care workers emphasize the importance of infection prevention and control measures. Moderate relative vaccine effectiveness with a waning trend reiterates the need for more efficacious vaccines against symptomatic infection caused by circulating variants and with longer duration of protection.

COVID-19, caused by severe acute respiratory syndrome coronavirus 2 (SARS-CoV-2), has resulted in substantial morbidity and mortality globally. (*1*) Before COVID-19 vaccines were developed and widely rolled out, various public health and social measures (PHSMs) were the only countermeasures to limit the spread of SARS-CoV-2 and thus were implemented as obligations or strong recommendations in each country. (*2*) Some of these PHSMs included lockdowns, mask mandates and border closures. Many studies have been conducted in various countries to evaluate the behavioural and social factors associated with SARS-CoV-2 infection to inform decision-making related to such PHSMs (*3*-*5*) However, such evidence is scarce in low- and middle-income countries (LMICs). Furthermore, once safe and effective vaccines were rolled out, concerns about waning immunity and the emergence of variants with immune escape capacity necessitated the monitoring of real-world vaccine effectiveness (VE). (*6*-*14*) There have been numerous studies to evaluate VE, mostly in high-income countries (HICs), but they have been limited in LMICs, including in Asia and the Pacific. (*15*) It would be valuable for more LMICs to conduct VE studies for the following reasons: (1) to evaluate vaccines that are mainly distributed in LMICs; (2) to confirm that the vaccines remain active through distribution networks, for example, no cold chain breaches; (3) to assemble data on the different cumulative infection burdens among countries, for example, to ascertain whether individuals with prior infection are protected against subsequent infection or disease; (4) to study substantial variations in PHSMs and policies or risk communication activities among countries; (5) to determine varied vaccine confidence within and among populations in surrounding countries; and (6) to build capacity to conduct operational research that would inform countries’ public health response to COVID-19 as well as future epidemics and pandemics.

In Japan, several authors from the present report previously evaluated behavioural factors associated with SARS-CoV-2 infection, many of which were in line with local policy or risk communication implementation, and estimated VE against symptomatic infection. (*5*, *14*, *16*-*18*) We used the same design (multicentre case-control study) to examine: (1) behavioural factors associated with SARS-CoV-2 infection; and (2) VE against symptomatic SARS-CoV-2 infection in the Philippines.

## Methods

### COVID-19 epidemiology and vaccination rollout in the Philippines

**Fig. 1:**
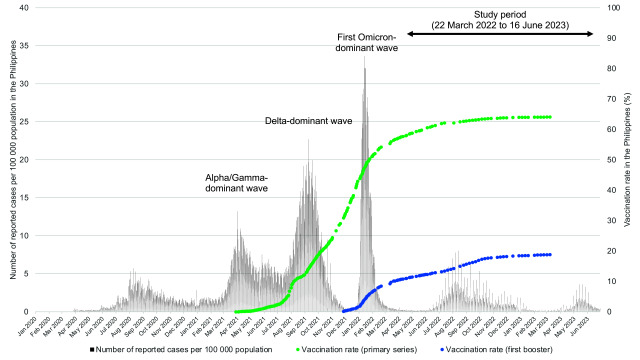
Number of reported COVID-19 cases since the beginning of the pandemic and COVID-19 vaccination rate with primary series and first booster, the Philippines

The epidemic curve of reported COVID-19 cases and vaccination rollout in the Philippines are illustrated together with the study period (22 March 2022 to 16 June 2023) in **Fig. 1**. In the Philippines, rollout of the primary series, that is, one vaccine dose from Janssen (J&J) or two doses of all other vaccine types, began on 1 March 2021. (*19*) The first booster dose rollout began on 16 November 2021 among health-care workers (HCWs), on 22 November 2021 among senior citizens and immunocompromised persons, and on 3 December 2021 among all adults aged ≥ 18 years. The second booster dose rollout began on 25 April 2022 among HCWs and individuals aged ≥ 60 years, and on 27 July 2022 among individuals aged ≥ 50 years and those aged 18–49 years with comorbidities. The primary series followed manufacturer-recommended intervals. During the study period from March 2022 to June 2023, Omicron subvariants B.1.1.529 and XBB.1.5 were reported to be dominant, while all the vaccines used were based on the ancestral strain, as variant-containing vaccines were not available at the time of the study. (*20*)

[**insert** Figure 1]

### Study design and setting

Our study, Factors Associated with SARS-CoV-2 INfection And The Effectiveness of COVID-19 vaccines in the Philippines (FASCINATE-P study), is a multicentre case-control study in health-care facilities with two objectives: (1) to elucidate behavioural and demographic risk factors associated with medically attended SARS-CoV-2 infection; and (2) to estimate the real-world effectiveness of COVID-19 vaccines used in the study country against symptomatic infection. This study was conducted at the Philippine General Hospital and San Lazaro Hospital in Manila, which had outpatient clinics that routinely tested individuals using polymerase chain reaction (PCR) for clinical diagnostic purposes and were functioning as two key COVID-19 response sites in the country. (*21*, *22*) We followed the same design as studies conducted in Japan and published previously. (*5*, *14*, *16*-*18*)

### Inclusion and exclusion criteria

All symptomatic individuals aged ≥ 18 years who sought care and had been tested for SARS-CoV-2 were included in the study. We defined symptomatic individuals as those with either fever ≥ 37.5 °C, malaise, chills, joint pain, headache, runny nose, cough, sore throat, shortness of breath, gastrointestinal symptoms (vomiting, diarrhoea or stomach ache), or loss of taste or smell. Individuals who did not or could not consent to participate in the study, individuals who required immediate life-saving treatment, and individuals who had previously participated in this study were excluded. At the analysis stage, we excluded individuals with unknown symptom onset date or who were tested ≥ 15 days after symptom onset.

### Classification of exposures and outcomes

Trained research nurses conducted face-to-face interviews before the PCR results were available to avoid social desirability bias, where individuals who tested positive were less likely to report potentially high-risk behaviours or more likely to report vaccination status. The interview collected general information (for example, sociodemographic factors) from the past  2 weeks relating to symptoms, preventive measures such as mask wearing, history of close contact, history of working or school attendance, history of behaviours such as social gatherings, and COVID-19 vaccination status. Patients were asked to present vaccination cards to ascertain the number of doses, vaccine manufacturer and date of each dose. Vaccination status was classified into 15 categories: (1) not vaccinated; (2) dose 1 or ≤ 13 days after dose 2 (partially vaccinated); (3) 14 days–3 months (14–90 days) after dose 2; (4) 3–6 months (90–180 days) after dose 2; (5) 6–9 months (181–270 days) after dose 2; (6) 9–12 months (271–360 days) after dose 2; (7) > 12 months (> 361 days) after dose 2; (8) ≤ 13 days after first booster dose; (9) 14 days–3 months (14–90 days) after first booster dose; (10) 3–6 months (90–180 days) after first booster dose; (11) > 6 months (> 181 days) after first booster dose; (12) ≤ 13 days after second booster dose; (13) 14 days–3 months (14–90 days) after second booster dose; (14) 3–6 months (90–180 days) after second booster dose; and (15) > 6 months  (> 181 days) after second booster dose.

SARS-CoV-2 PCR was carried out at each medical facility for diagnostic purposes; PCR-positive individuals were considered cases, and PCR-negative individuals were controls.

### Sample size calculation

For risk factor analysis, assuming 10% positivity (based on data when the study was planned), 30–50% of controls with exposure of interest, a two-tailed significance level of 5%, and 80% power, enrolment of approximately 70–80 cases and 700–800 controls was needed for a minimum detectable odds ratio of 2. For VE estimates, assuming 10% positivity, expected vaccine coverage of 30% and 90% VE (based on data from the ancestral strain when the study was planned), 207 cases and 1864 controls were needed for the lower confidence interval (CI) boundary of 10%. We planned to continue enrolment even after reaching this target to allow for subanalysis and continued assessment of factors that may be time-varying.

### Data analysis

Participant characteristics and vaccination status were described.

For risk factor analysis, individuals with a history of close contact were excluded because an infection, if confirmed, is usually most likely due to this specific contact rather than exposures solicited in the questionnaire. Logistic regression to identify associations between behavioural risk factors and SARS-CoV-2 infection was conducted, adjusting for age, sex, presence of comorbidities, prior SARS-CoV-2 infection, testing date (one categorical variable for every 2 weeks, for example, weeks 41–42 of 2022 as one variable), study site and vaccination status by dosage. These potential confounders were determined a priori based on published reports. (*5*)

For VE evaluation, to reduce confounding by various socioeconomic factors and priority of vaccination that can be confounders, we restricted the analyses to HCWs, older adults and individuals with comorbidities (who were also eligible for the fourth dose). Logistic regression was used to estimate the odds of being vaccinated among cases relative to controls. The model was adjusted for age, sex, presence of comorbidities, history of close contact, SARS-CoV-2 testing in the past month, prior SARS-CoV-2 infection, education, working or school attendance, going out to eat or drink in the evening/night without alcohol, testing date (one categorical variable for every two weeks, for example, weeks 41–42 of 2022 as one variable) and study site. These potential confounders were also determined a priori based on published reports. (*14*) VE against medically attended symptomatic SARS-CoV-2 infection was estimated using the following equation: VE = (1 – aOR) × 100%. In addition to absolute VE (aVE; VE comparing the vaccinated and unvaccinated), we planned to calculate relative VE (rVE; VE comparing individuals who received a booster of interest vs individuals who only received the previous dose 3 or more months earlier, for example, VE comparing three vs two doses and VE comparing four doses vs three doses) to evaluate the added effect of the booster.

Data analyses were performed using STATA version 18.0.

### Choice of controls in risk factor analysis

We considered that the behavioural and demographic traits among cases and controls would be most similar, as they were sourced from those presenting to the same medical facilities for testing (for example, health-seeking behaviours). Also, if controls were infected with other viruses due to similar exposures, the odds ratio for SARS-CoV-2 infection would be an underestimate of the true association. In other words, our design would detect differences in the magnitude of a particular risk factor or risk factors that would be specific to COVID-19. In fact, even though many respiratory pathogens (influenza virus, *Streptococcus pneumoniae*, etc.) were circulating at extremely low levels during the early phase of the pandemic, possibly due to PHSMs, SARS-CoV-2 epidemics occurred repeatedly. This suggests that SARS-CoV-2 has unique features that allow it to circulate even under strict PHSMs Please see the Supplementary Methods of our previous report (*5*) for further detailed rationale.

## Results

### Characteristics of the study participants

**Fig. 2:**
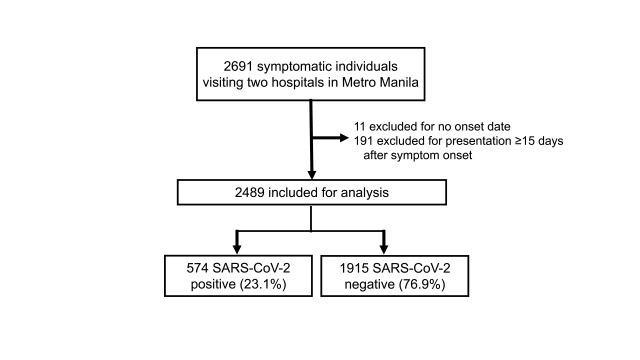
Flow diagram of the multicentre case-control study participants, the Philippines

A total of 2691 symptomatic individuals were enrolled from two hospitals during the study period; we excluded 11 individuals due to unknown symptom onset date and 191 due to being tested ≥ 15 days after symptom onset (**Fig. 2**). The final analysis included 2489 individuals with 574 (23.1%) positive cases. The median interquartile age range (IQR) was 35 (27–51), 892 (35.8%) were male, and 877 (35.2%) had comorbidities (**Table 1**); 1743 (70.1%) were working. Although data on race and ethnicity were not collected, 2486 (99.9%; three missing) were Filipinos. All participants answered that they wore a mask when going out. Most had received COVID-19 vaccines (2246, 90.2%). Among the vaccine recipients, most had their vaccination cards (2123, 94.5%). Among those vaccinated with the primary series, 39% received AstraZeneca, 37% received Sinovac, 11% received Pfizer, 7% received Moderna, and 6% received other types. Among the recipients of booster doses, over 90% received mRNA vaccines.

[**insert** Figure 2]

**Table 1:** Multicentre case-control study: demographic and clinical characteristics of participants, the Philippines

Characteristic	All (*n* = 2 489)	Test positive (*n* = 574)	Test negative (*n* = 1915)
**Age in years, *n*(%)**	**35 (27–51)a**	**32 (26–43)a**	**37 (28–52)a**
**18–19**	**50 (2.0)**	**16 (2.8)**	**34 (1.8)**
**20–29**	**830 (33.4)**	**239 (41.6)**	**591 (30.8)**
**30–39**	**594 (23.9)**	**158 (27.5)**	**436 (22.8)**
**40–49**	**352 (14.1)**	**69 (12.0)**	**283 (14.8)**
**50–59**	**359 (14.4)**	**62 (10.8)**	**297 (15.5)**
**60–69**	**194 (7.8)**	**24 (4.2)**	**170 (8.9)**
**70–79**	**98 (3.9)**	**6 (1.1)**	**92 (4.8)**
**80–89**	**12 (0.5)**	**0 (0.0)**	**12 (0.6)**
**Sex,** *n* **(%)**			
**Male**	**892 (35.8)**	**178 (31.0)**	**714 (37.3)**
**Female**	**1597 (64.2)**	**396 (69.0)**	**1201 (62.7)**
**Educational attainment,** *n* **(%)**			
**Master’s degree and above**	**158 (6.4)**	**51 (8.9)**	**107 (5.6)**
**College**	**1570 (63.1)**	**458 (79.8)**	**1112 (58.1)**
**Vocational**	**128 (5.1)**	**18 (3.1)**	**110 (5.7)**
**Secondary/high school**	**526 (21.1)**	**41 (7.1)**	**485 (25.3)**
**Primary/elementary**	**107 (4.3)**	**6 (1.1)**	**101 (5.3)**
**Comorbidity,^b^** *n* **(%)**			
**Yes**	**877 (35.2)**	**126 (22.0)**	**751 (39.2)**
**No**	**1612 (64.8)**	**448 (78.1)**	**1164 (60.8)**
**Occupation,** *n* **(%)**	** -**	** -**	**-**
**Health-care worker**	**1207 (48.5)**	**400 (69.7)**	**807 (42.1)**
**Other**	**1282 (51.5)**	**174 (30.3)**	**1108 (57.9)**
**Smoking,** *n* **(%); missing = 7 (0.3%)**			
**Never smoked**	**2042 (82.3)**	**520 (90.8)**	**1522 (79.7)**
**Past smoker**	**346 (13.9)**	**35 (6.1)**	**311 (16.3)**
**Current smoker**	**94 (3.8)**	**18 (3.1)**	**76 (4.0)**
**Days from onset to SARS-CoV-2 test**	**3 (2–5)**	**2 (2–3)**	**3 (2–6)**
**History of close contact,** *n* **(%)**			
**Yes**	**401 (16.1)**	**149 (26.0)**	**252 (13.2)**
**No/unknown**	**2088 (83.9)**	**425 (74.0)**	**1663 (86.8)**
**SARS-CoV-2 diagnostic test in the past month,** *n* **(%); missing = 1 (0.0%)**			
**Yes**	**599 (22.5)**	**94 (16.4)**	**465 (24.3)**
**No**	**1929 (77.5)**	**480 (83.6)**	**1449 (75.7)**
**Past SARS-CoV-2 infection,** *n* **(%)**			
**No**	**1801 (72.4)**	**395 (68.8)**	**1406 (73.4)**
**Once**	**627 (25.2)**	**164 (28.6)**	**463 (24.2)**
**Twice**	**57 (2.3)**	**13 (2.3)**	**44 (2.3)**
**Three times**	**4 (0.2)**	**2 (0.4)**	**2 (0.1)**
**Vaccination card carrying,** *n* **(%)**	** -**	** -**	** -**
**Yes**	**2123 (94.5)**	**532 (94.7)**	**1591 (94.5)**
**No**	**123 (5.5)**	**30 (5.3)**	**93 (5.5)**
**Number of COVID-19 vaccinations received,** *n* **(%)**			
**None**	**243 (9.8)**	**12 (2.1)**	**231 (12.1)**
**Once (except for Ad26.COV2.S^c^)**	**15 (0.6)**	**2 (0.4)**	**13 (0.7)**
**Twice or received Ad26.COV2.S**	**682 (27.4)**	**76 (13.2)**	**606 (31.6)**
**First booster received**	**820 (32.9)**	**232 (40.4)**	**588 (30.7)**
**Second booster received**	**729 (29.3)**	**252 (43.9)**	**477 (24.9)**
**Vaccine type (primary series),** *n* **(%)**			
**AZD1222 (AstraZeneca)**	**868 (38.6)**	**265 (47.2)**	**603 (35.8)**
**CoronaVac (Sinovac)**	**828 (36.9)**	**187 (33.3)**	**641 (38.1)**
**BNT162b2 (Pfizer)**	**249 (11.1)**	**46 (8.2)**	**203 (12.1)**
**mRNA-1273 (Moderna)**	**159 (7.1)**	**40 (7.1)**	**119 (7.1)**
**Ad26.COV2.S (Janssen/J&J)**	**50 (2.2)**	**6 (1.3)**	**44 (2.6)**
**Sputnik V (Gameleya)**	**41 (1.8)**	**7 (1.3)**	**34 (2.0)**
**BBIBP-CorV (Sinopharm)**	**7 (0.3)**	**0 (0.0)**	**7 (0.4)**
**BBV152 (Bharat BioTech)**	**1 (0.0)**	**0 (0.0)**	**1 (0.1)**
**Unknown**	**1 (0.0)**	**1 (0.2)**	**0 (0.0)**
**Heterologous**	**42 (1.9)**	**10 (1.8)**	**32 (1.9)**
**Vaccine type (first booster),** *n* **(%)**			
**BNT162b2 (Pfizer)**	**1149 (74.2)**	**381 (78.7)**	**768 (72.1)**
**mRNA-1273 (Moderna)**	**250 (16.1)**	**67 (13.8)**	**183 (17.2)**
**AZD1222 (AstraZeneca)**	**109 (7.0)**	**26 (5.4)**	**83 (7.8)**
**CoronaVac (Sinovac)**	**39 (2.5)**	**10 (2.1)**	**29 (2.7)**
**Ad26.COV2.S (Janssen/J&J)**	**1 (0.1)**	**0 (0.0)**	**1 (0.1)**
**Sputnik V (Gameleya)**	**1 (0.1)**	**0 (0.0)**	**1 (0.1)**
**Vaccine type (second booster),** *n* **(%)**	**-**	** -**	** -**
**BNT162b2 (Pfizer)**	**407 (55.8)**	**141 (56.0)**	**266 (55.8)**
**mRNA-1273 (Moderna)**	**315 (43.2)**	**111 (44.1)**	**204 (42.8)**
**AZD1222 (AstraZeneca)**	**6 (0.8)**	**0 (0.0)**	**6 (1.3)**
**Sputnik V (Gameleya)**	**1 (0.1)**	**0 (0.0)**	**1 (0.2)**

*n*: number.

^a^ Median (interquartile range).

^b^ Comorbidities (self-reported) include hypertension, heart disease, diabetes mellitus, kidney disease, asthma, chronic obstructive pulmonary disease, obesity, cancer, immunodeficiency and immunosuppressant use.

^c^ Primary series is one dose, whereas other vaccine types are two doses.

### Association between sociobehavioural factors and medically attended SARS-CoV-2 infection

After excluding individuals with a history of close contact, 2088 individuals were included in this analysis. No apparent association was observed between SARS-CoV-2 infection and socioeconomic factors such as cohabitation status, education or household income (**Table 2**). On the other hand, interviewees who were working or attending school, especially HCWs, were associated with SARS-CoV-2 infection, with an adjusted odds ratio (aOR) of 1.83 (95% CI: 1.09–3.07) for those working or in school and an aOR of 1.45 (95% CI: 1.03–2.06) specifically for HCWs. No apparent association was observed between SARS-CoV-2 infection and various social gatherings with food or drinks, except for a statistically nonsignificant trend of higher infection risk among those who went out to eat or drink in the evening/night without alcohol (1.31 [0.94–1.82]; therefore, this was included as one of the covariates for the VE analysis. However, among those who attended social gatherings, the odds of infection were higher among individuals who attended gatherings of five or more people compared to those who attended smaller gatherings (aOR: 2.58, 95% CI: 1.14–5.83). They were also higher among individuals who attended gatherings that lasted 2 hours or longer compared to individuals who attended shorter gatherings (aOR: 1.75, 95% CI: 0.95–3.22). The odds of infection were not higher among those who ordered takeaway, used food-delivery services or ate out alone compared to those who did not. Other behaviours unrelated to food or drink were also not apparently associated with SARS-CoV-2 infection, except that the odds of infection were slightly higher among those who reported having gone to the gym (aOR: 1.53, 95% CI: 0.94–2.49) or to karaoke (aOR: 1.74, 95% CI: 0.81–3.86) (**Table 2**).

**Table 2:** Multicentre case-control study: association between sociobehavioural factors and SARS-CoV-2 infection, the Philippines

Sociobehavioural factors	Test positive, *n*(%)	Test negative, *n*(%)	Crude odds ratios (95% CI)	Adjusted odds ratios (95% CI)^a^
**Cohabitation**				
**Living alone**	**87 (28.9)**	**214 (71.1)**	**1**	**1**
**Living with family**	**244 (16.1)**	**1276 (84.0)**	**0.47 (0.35–0.63)**	**0.86 (0.61–1.24)**
**Living with people other than family**	**94 (35.2)**	**173 (64.8)**	**1.34 (0.94–1.90)**	**1.12 (0.74–1.70)**
**Education**				
**Primary/elementary**	**6 (5.6)**	**101 (94.4)**	**1**	**1**
**Secondary/high school**	**38 (7.4)**	**475 (92.6)**	**1.35 (0.55–3.27)**	**0.89 (0.34–2.34)**
**Vocational**	**16 (13.7)**	**101 (86.3)**	**6.22 (2.71–14.31)**	**1.30 (0.48–3.50)**
**College**	**343 (27.0)**	**928 (73.0)**	**6.39 (2.45–16.65)**	**1.12 (0.35–3.54)**
**Post-graduate/master’s degree/PhD**	**22 (27.5)**	**58 (72.5)**	**2.67 (1.00–7.09)**	**1.20 (0.39–3.71)**
**Monthly household income**				
**Unemployed/no income**	**5 (2.9)**	**166 (97.1)**	**1**	**1**
**<** ₱**10 000 (< US$ 176.50)**	**17 (6.1)**	**261 (93.9)**	**2.16 (0.78–5.97)**	**0.93 (0.25–3.51)**
₱**10 000– < 50 000 (US$ 176.50–882.60)**	**169 (18.4)**	**748 (81.6)**	**7.50 (3.03–18.54)**	**1.08 (0.29–3.97)**
₱**50 000– < 80 000 (US$ 882.60–1412.20)**	**150 (34.1)**	**290 (65.9)**	**17.17 (6.90–42.71)**	**1.31 (0.34–5.06)**
**≥** ₱**80 000 (≥ US$ 1 412.20)**	**78 (34.8)**	**146 (65.2)**	**17.74 (6.99–45.00)**	**1.39 (0.35–5.47)**
**Work or school attendance**				
**No**	**49 (6.7)**	**682 (93.3)**	**1**	**1**
**Yes**	**376 (27.7)**	**978 (72.2)**	**5.35 (3.91–7.32)**	**1.83 (1.09–3.07)**
**Health-care worker**				
**No**	**153 (12.7)**	**1055 (87.3)**	**1**	**1**
**Yes**	**272 (30.9)**	**608 (69.1)**	**3.08 (2.47–3.85)**	**1.45 (1.03–2.06)**
**Going out to eat/drink in the daytime with alcohol**				
**No**	**422 (20.4)**	**1646 (79.6)**	**1**	**1**
**Yes**	**3 (15.0)**	**17 (85.0)**	**0.69 (0.20–2.36)**	**0.36 (0.09–1.38)**
**Going out to eat/drink in the evening/night with alcohol**				
**No**	**393 (19.9)**	**1585 (80.1)**	**1**	**1**
**Yes**	**32 (29.1)**	**78 (70.9)**	**1.65 (1.08–2.53)**	**1.24 (0.74–2.06)**
**Going out to eat/drink in the daytime without alcohol**				
**No**	**259 (16.4)**	**1322 (83.6)**	**1**	**1**
**Yes**	**166 (32.7)**	**259 (16.4)**	**2.48 (1.98–3.12)**	**0.90 (0.64–1.25)**
**Going out to eat/drink in the evening/night without alcohol**				
**No**	**296 (17.3)**	**1421 (82.8)**	**1**	**1**
**Yes**	**129 (34.8)**	**296 (17.2)**	**2.56 (2.00–3.28)**	**1.31 (0.94–1.82)**
**Going to a café**				
**No**	**346 (19.5)**	**1425 (80.5)**	**1**	**1**
**Yes**	**79 (24.9)**	**238 (75.1)**	**1.37 (1.03–1.81)**	**0.97 (0.69–1.35)**
**Maximum number of people who attended the gatherings with food/drinks including oneself within 2 weeks of onset**				
** < 5 people**	**65 (22.9)**	**219 (77.1)**	**1**	**1**
** ≥ 5 people**	**15 (44.1)**	**19 (55.9)**	**2.66 (1.28–5.53)**	**2.58 (1.14–5.83)**
**Maximum time spent at the gatherings with food/drinks attended within 2 weeks of onset**				
** < 2 hours**	**27 (17.7)**	**126 (82.4)**	**1**	**1**
** ≥ 2 hours**	**53 (32.3)**	**111 (67.7)**	**2.23 (1.31–3.78)**	**1.75 (0.95–3.22)**
**Ordering takeaway**				
**No**	**290 (21.3)**	**1075 (78.8)**	**1**	**1**
**One**	**13 (22.8)**	**44 (77.2)**	**1.10 (0.58–2.06)**	**1.12 (0.52–2.39)**
**Twice**	**59 (21.0)**	**222 (79.0)**	**0.99 (0.72–1.35)**	**1.14 (0.78–1.67)**
**Three times or more**	**63 (16.4)**	**322 (83.6)**	**0.72 (0.54–0.98)**	**0.98 (0.68–1.40)**
**Using food delivery**				
**No**	**215 (15.3)**	**1187 (84.6)**	**1**	**1**
**One**	**5 (9.8)**	**46 (90.2)**	**0.60 (0.24–1.53)**	**0.34 (0.12–0.93)**
**Twice**	**35 (27.6)**	**92 (72.4)**	**2.10 (1.39–3.18)**	**1.10 (0.67–1.80)**
**Three times or more**	**170 (33.5)**	**338 (66.5)**	**2.78 (2.20–3.51)**	**1.18 (0.85–1.62)**
**Eating out alone**				
**No**	**410 (20.6)**	**1579 (79.4)**	**1**	**1**
**Yes**	**15 (15.2)**	**84 (84.9)**	**0.69 (0.39–1.20)**	**0.81 (0.43–1.53)**
**Going to a mall**				
**No**	**148 (14.4)**	**878 (85.6)**	**1**	**1**
**Yes**	**277 (26.1)**	**785 (73.9)**	**2.09 (1.68–2.61)**	**1.07 (0.80–1.42)**
**Going to a gym**				
**No**	**390 (19.8)**	**1578 (80.2)**	**1**	**1**
**Yes**	**35 (29.2)**	**85 (70.8)**	**1.67 (1.11–2.51)**	**1.53 (0.94–2.49)**
**Going to karaoke**				
**No**	**411 (20.1)**	**1635 (79.9)**	**1**	**1**
**Yes**	**14 (33.3)**	**28 (66.7)**	**1.99 (1.03–3.81)**	**1.76 (0.81–3.86)**
**Going to church**				
**No**	**308 (22.4)**	**1069 (77.6)**	**1**	**1**
**Yes**	**117 (16.5)**	**594 (83.5)**	**0.68 (0.54–0.86)**	**0.89 (0.66–1.20)**

₱: Philippine peso; CI: confidence interval; *n*: number; PhD: Doctor of Philosophy; US$: US dollar.

^a^ Adjusted for age, sex, comorbidities, prior infection, week of testing, study site and vaccine by dosage.

### Association between COVID-19 vaccination (by doses and period since vaccination) and medically attended SARS-CoV-2 infection

After restricting to HCWs, older adults and individuals with comorbidities, 1890 individuals were included in this analysis. In the comparison between vaccinated and unvaccinated individuals, there were inconsistent odds of infection depending on the vaccination category. In the comparison between the first booster and 3 months after the primary series, there was a moderate effect 14 days to 3 months after the booster dose (rVE: 32%, 95% CI: −120–79), but VE seems to wane after half a year (rVE: −8%, 95% CI: −72–33). The comparison between the second booster and 3 months after the first booster showed a similar trend of moderate effect in the short term (rVE: 48%, 95% CI: −23–78) with waning protection (**Table 3**).

**Table 3:** Multicentre case-control study: association between COVID-19 vaccination (by doses and time since vaccination) and SARS-CoV-2 infection, the Philippines

Vaccination status	Test positive	Test negative	Crude odds ratios (95% CI)^a^	Adjusted odds ratios (95% CI)^a^	VE% (95% CI)
**Comparison between vaccinated and unvaccinated**					
**Unvaccinated**	**11**	**171**	**1**	**1**	**N/A**
**Dose 1 or ≤ 13 days after primary series**	**2**	**11**	**2.83 (0.56–14.36)**	**2.08 (0.35–12.4)**	**Not calculated^b^**
**14 days to 3 months after primary series**	**0**	**12**	**N/A**	**N/A**	**Not calculated^b^**
**3–6 months after primary series**	**2**	**42**	**0.74 (0.16–3.47)**	**0.69 (0.13–3.59)**	**Not calculated^b^**
**6–9 months after primary series**	**6**	**73**	**1.28 (0.46–3.59)**	**0.78 (0.25–2.42)**	**Not calculated^b^**
**9–12 months after primary series**	**17**	**114**	**2.32 (1.05–5.13)**	**2.57 (1.06–6.19)**	**Not calculated^b^**
** > 12 months after primary series**	**29**	**157**	**2.87 (1.39–5.94)**	**1.43 (0.59–3.50)**	**Not calculated^b^**
** ≤ 13 days after first booster**	**0**	**0**	**N/A**	**N/A**	**Not calculated^b^**
**14 days to 3 months after first booster**	**5**	**23**	**3.38 (1.08–10.60)**	**0.96 (0.25–3.64)**	**Not calculated^b^**
**3–6 months after first booster**	**12**	**69**	**2.70 (1.14–6.42)**	**1.07 (0.38–3.02)**	**Not calculated^b^**
** > 6 months after first booster**	**160**	**348**	**7.15 (3.78–13.52)**	**1.57 (0.66–3.72)**	**Not calculated^b^**
** ≤ 13 days after second booster**	**2**	**3**	**10.36 (1.57–68.6)**	**2.94 (0.35–24.55)**	**Not calculated^b^**
**14 days to 3 months after second booster**	**8**	**31**	**4.01 (1.49–10.77)**	**0.77 (0.24–2.50)**	**Not calculated^b^**
**3–6 months after second booster**	**78**	**153**	**7.93 (4.06–15.45)**	**1.46 (0.59–3.59)**	**Not calculated^b^**
** > 6 months after second booster**	**121**	**230**	**8.18 (4.28–15.64)**	**2.05 (0.83–5.09)**	**Not calculated^b^**
**Comparison between the first booster and 3 months after primary series**					
** > 3 months after primary series**	**54**	**386**	**1**	**1**	**N/A**
** ≤ 13 days after first booster**	**0**	**0**	**N/A**	**N/A**	**N/A**
**14 days to 3 months after first booster**	**5**	**23**	**1.55 (0.57–4.26)**	**0.68 (0.21–2.20)**	**32 (−120–79)**
**3–6 months after first booster**	**12**	**69**	**1.24 (0.63–2.44)**	**0.73 (0.33–1.60)**	**27 (−60–67)**
** > 6 months after first booster**	**160**	**348**	**3.29 (2.34–4.62)**	**1.08 (0.67–1.72)**	**-8 (−72–33)**
**Comparison between the second booster and 3 months after the first booster**					
** > 3 months after first booster**	**172**	**417**	**1**	**1**	**N/A**
** ≤ 13 days after second booster**	**2**	**3**	**1.62 (0.27–9.76)**	**1.96 (0.27–14.0)**	**Too few**
**14 days to 3 months after second booster**	**8**	**31**	**0.63 (0.28–1.39)**	**0.52 (0.22–1.23)**	**48 (−23–78)**
**3–6 months after second booster**	**78**	**153**	**1.24 (0.89–1.71)**	**0.98 (0.66–1.43)**	**2 (−43–34)**
** > 6 months after second booster**	**121**	**230**	**1.28 (0.96–1.69)**	**1.34 (0.94–1.91)**	**-34 (−91–6)**

CI: confidence interval; VE: vaccine effectiveness; N/A: not applicable; d: day; mo: month.

^a^ Adjusted for age, sex, comorbidities, history of close contact, SARS-CoV-2 testing in the past month, prior infection, education, work/school, going out to eat/drink in the evening/night without alcohol, week of testing, study site.

^b^ Not calculated due to high risk of bias.

## Discussion

In this multicentre case-control study in the Philippines, we investigated the association between various sociobehavioural factors and medically attended SARS-CoV-2 infection. We also examined the association between COVID-19 vaccination and medically attended SARS-CoV-2 symptomatic infection. By following the same design as a similar study conducted in Japan by some of the authors, we aimed to look at country-specific differences in factors associated with SARS-CoV-2 infection. (*5*)

First, there was no apparent association between socioeconomic factors such as cohabitation status, education or household income and SARS-CoV-2 infection, suggesting that SARS-CoV-2 has spread regardless of socioeconomic status. However, working, especially in the health-care environment, had higher odds of SARS-CoV-2 infection compared to not working or not working in the health-care environment, respectively. This was also observed in other countries early in the pandemic. (*23*) With proper personal protective equipment (PPE) and infection prevention and control measures in the health-care setting, the risk of occupational exposure should have been minimized, but this trend was not observed in Japan, where strict infection prevention and control measures were in place. (*5*, *24*) Policies should also make sure that adequate supplies of PPE are available to protect those on the front line. We next examined various behaviours that may be associated with SARS-CoV-2 infection. Among those who attended social gatherings, the odds of infection were higher among individuals who attended gatherings of five or more people compared to smaller gatherings and individuals who attended for 2 hours or longer compared to shorter durations. Although not statistically significant, going to the gym or karaoke may be associated with higher odds of infection, while other behaviours such as ordering takeaway, using food-delivery services and eating out alone were not associated with infection. These findings were in line with findings from Japan and highlighted the nature of this pathogen where transmission can occur efficiently in specific situations. (*5*, *25*)

We examined the association between COVID-19 vaccination and medically attended SARS-CoV-2 infection to estimate COVID-19 VE against symptomatic infection. As for the comparison between vaccinated and unvaccinated individuals, there were inconsistent odds of infection depending on the vaccination category. We did include various covariates in the multivariable analysis, but we suspected that the risk of residual bias was high and, therefore, aVE was not presented. One bias that could have caused this is that, due to a substantial delay in the ethics approval process, enrolment began after a large Omicron wave in early 2022, when the majority of unvaccinated individuals were already or recently infected without having been tested, resulting in a protective effect at a level higher than that from vaccination several months earlier. Also, the presentation of vaccination cards was required in some stores and restaurants, which could have potentially underestimated VE. (*18*) This is in line with reports from Canada, where negative effectiveness was observed. (*26*, *27*) On the other hand, moderate rVE for the first booster (32%) and the second booster (48%) against medically attended symptomatic SARS-CoV-2 infection was observed (although neither was statistically significant due to the small sample size). However, these effects seemingly have waned after half a year. These findings were consistent with the Japanese study and studies from other countries (*10*-*17*) and reiterate the need for vaccines that are more effective against symptomatic infection caused by circulating variants and with a longer duration of protection.

### Limitations

This study had several limitations. First, biases inherent in observational studies are possible. Using a detailed questionnaire, we attempted to minimize confounding that is not necessarily accounted for in studies that retrospectively evaluate routine surveillance data, but unmeasured and residual confounding could have occurred. However, as explained above, the association between vaccination and medically attended SARS-CoV-2 infection has probably had residual bias with most unvaccinated individuals being infected, and thus aVE was not presented. Second, for the risk factor analyses, controls may have been infected with other viruses due to similar exposures, which can underestimate the odds ratio (see Methods for details). Third, identified risk factors may be country-, region-, culture- and population-specific and time-dependent due to changes in COVID-19-related policies and behaviours. Also, the determination of past infection was likely suboptimal, and this could have protected “truly high-risk groups” from getting infected during the study period. Specifically, our study population had a large proportion of HCWs, and thus the risk factor analyses may not be generalizable to the overall population in the Philippines. Fourth, our primary analyses were complete case analyses. However, due to the prospective nature of the study with thorough interviews, the amount of missing data was minimal, as shown in **Table 1**. Fifth, some estimates were calculated based on very low numbers, resulting in wide CIs that warrant careful interpretation. Finally, the study sites were two hospitals, which may limit the generalizability to the whole country.

## Conclusions

In this case-control study in the Philippines, school attendance or working, especially in the health-care environment, had higher odds of SARS-CoV-2 infection compared to not working or not working in the health-care environment, respectively, suggesting the importance of infection prevention and control measures in the health-care setting. Also, attending social gatherings with five or more people or for a longer duration was associated with SARS-CoV-2 infection. Although a comparison of COVID-19 VE versus unvaccinated groups could not be estimated due to the high risk of bias, moderate rVE against symptomatic SARS-CoV-2 infection was observed, albeit with a waning trend after half a year.
